# Case Report: *HAVCR2* mutation-associated Hemophagocytic lymphohistiocytosis

**DOI:** 10.3389/fimmu.2023.1271324

**Published:** 2023-11-23

**Authors:** Deli Song, Jingshi Wang, Jia Zhang, Junxia Hu, Chaofan Wu, Zhao Wang

**Affiliations:** Department of Hematology, Capital Medical University Affiliated Beijing Friendship Hospital, Beijing, China

**Keywords:** HAVCR2 mutation, hemophagocytic lymphohistiocytosis, immunodeficiency, subcutaneous panniculitis-like T-cell lymphoma, cytokine

## Abstract

Germline *HAVCR2* mutation has been reported to be associated with subcutaneous panniculitis-like T-cell lymphoma (SPTCL) leading to Hemophagocytic lymphohistiocytosis (HLH). Several studies have indicated that *HAVCR2* mutation can cause HLH even in the absence of lymphoma, though the exact mechanism remains unclear. In this article, we reported five cases of *HAVCR2* mutation-associated HLH. Our analysis revealed an elevated level of IL-1RA in the serum of these patients. Furthermore, we investigated the potential mechanisms underlying HLH associated with *HAVCR2* mutation based on changes in cytokine levels. Our findings suggest that *HAVCR2* mutation may represent a distinct genetic defect underlying HLH, differing from traditional primary HLH.

## Introduction

Hemophagocytic lymphohistiocytosis (HLH) is a highly inflammatory disease involving cytokine storms and is characterized by rapid progression and high mortality. It is classified into primary and secondary hemophagocytic syndromes (1). Classical primary HLH-associated gene is involved in impaired cytotoxic function (e.g., *PRF1*, *UNC13D*, *STXBP2*, and *STX11*), aberrant inflammasome regulation (e.g., *NLRC4*), and increased susceptibility to Epstein-Barr virus infection (e.g.,*CD27* and *CD70*) (2). Furthermore, it has been discovered that various genes linked to compromised pathogen control, dysregulated activation of inflammasome, and metabolic disorders are also connected to the occurrence of HLH (2, 3). Germline *HAVCR2* mutation is associated with HLH in patients with subcutaneous panniculitis-like T-cell lymphoma (SPTCL) due to the decreased expression of T cell immunoglobulin and mucin-containing molecule 3 (TIM-3) encoded by *HAVCR2* on various immune cells (4, 5). However, the poor prognosis of SPTCL-associated HLH carrying germline *HAVCR2* mutation is increasingly questioned, despite the frequent occurrence of *HAVCR2* mutation in SPTCL patients (6). Boonyabaramee et al. found that the prognosis of *HAVCR2* mutation-associated HLH is relatively good (7), but exact mechanism is unclear. This report presents five cases of *HAVCR2* mutation-associated HLH that were attended to our center since January 2021. Furthermore, we analyzed the cytokine levels in these patients and speculated the potential mechanisms underlying HLH associated with *HAVCR2* mutation.

## Case series

### Case 1

A 39-year-old woman presented with 18-month history of fever and nodular rash. Examination revealed elevated transaminase levels and multiple enlarged lymph nodes. The pathology of the lymph nodes and subcutaneous nodules indicated necrotizing lymphadenitis and panniculitis, respectively. Treatment with methylprednisolone in the local hospital was ineffective, resulting in oedema, which was misdiagnosed as adult-onset Still’s disease. Following treatment with ruxolitinib, methylprednisolone, gamma globulin, and interleukin-6 monoclonal antibody, the patient’s temperature normalized, but the subcutaneous nodules did not improve. In addition, the fever later recurred, and the patient presented to our hospital. Blood examination showed liver dysfunction, cytopenia, poor coagulation, and increased levels of ferritin (52445.30 ng/mL) and soluble interleukin-2 receptor (sCD25) (107215 pg/mL). Following a repeat skin biopsy, the pathological diagnosis was SPTCL. Whole exon sequencing (WES) identified a homozygous mutation c245A>G (p.Y82C) in the *HAVCR2* gene, while her parents were found to be heterozygous for this mutation. Following treatment with a combination of ruxolitinib and the DEP regimen (liposomal doxorubicin, etoposide, and methylprednisolone) (8), the patient experienced relief from HLH and subsequently underwent lymphoma treatment. At the 16-month follow-up, the patient responded well to treatment. Additional details about the patient are shown in **Table 1**.

**Table 1:** Clinical characteristics of the patients at the onset of HLH.

	Case 1	Case 2	Case 3	Case 4	Case 5
Age at onset, year	39	30	39	39	33
Sex	Female	Male	Female	Female	Female
Fever, temperature >38.5°C	Y	Y	Y	Y	Y
WBC, 10^9^/L	0.73	0.73	4.67	19.75	2.0
HGB, g/L	84	102	106	93	96
PLT, 10^9^/L	61	78	81	50	81
Fbg, g/L	1.14	0.47	0.4	1.13	NA
TG, mmol/L	6.93	3.55	10.69	1.54	NA
ALT, U/L	183	116	414	NA	NA
Total bilirubin, μmol/L	54.66	15.09	70.00	NA	NA
Ferritin, ng/mL	52445.30	15138.40	839.10	15084	1682
sCD25, pg/mL	107215	7369	60585	6087	2269
NK cell activity, %	14.54	14.15	51.06	9.99	2.28
Hemophagocytosis	Y	Y	Y	Y	Y
Splenomegaly	Y	Y	Y	Y	Y
Subcutaneous nodules	Y	N	Y	Y	Y
SPTCL	Y	N	N	N	Y

WBC, white blood cell count (normal 3.5–9.5 × 10^9^/L); HGB, haemoglobin (normal 115–150 g/L); PLT, platelet count (normal 125–350 × 10^9^/L); Fbg, fibrinogen (normal 1.70–4.00 g/L); TG, triglyceride (normal 0.57–1.70 mmol/L); ALT, alanine transaminase (normal 7–40 U/L); Total bilirubin (normal 3.42–17.10 μmol/L); Ferritin (normal 24–336 ng/mL); NK cell activity (normal >15.11%). sCD25, soluble interleukin-2 receptor (normal <6,400 pg/mL). SPTCL, subcutaneous panniculitis-like T-cell lymphoma; Y, yes; N, No; NA, not available.

### Case 2

A 30-year-old man developed fever following a cold 3 months prior, accompanied by dry cough, general fatigue, and expectoration. The patient was administered piperacillin/tazobactam at another hospital, but fever did not improve. Laboratory tests revealed decreased white blood cell and platelet counts, as well as abnormal liver function and elevated levels of serum triglyceride (3.55mmol/L), ferritin (15138.40ng/mL), and sCD25(7369pg/mL). Bone marrow examination revealed hemophagocytosis. Positron emission tomography/computed tomography (PETCT) indicated an increase in the density of metabolically active fat in various regions including the bilateral supraclavicular region, bilateral cervical roots, peritoneum, and mesentery, as well as an enlarged spleen. He was diagnosed with HLH and treated with the DEP regimen; however, a slight fever persisted. The patient was admitted to our hospital, and no subcutaneous nodules were found on physical examination. The patient exhibited impaired hepatic function and developed cytopenia. Additionally, his ferritin and sCD25 levels were elevated, while his fibrinogen levels had decreased (**Table 1**). WES identified a homozygous mutation in *HAVCR2*, specifically c245A > G (p.Y82C), while the patient’s parents were found to be heterozygous for this mutation. These findings indicated a relapse of HLH. To address this, the patient was administered methylprednisolone and ruxolitinib. After two weeks, the patient achieved complete remission, as per the criteria proposed by Marsh et al. (9). Over the course of 12 months of follow-up, the patient’s condition stabilized.

### Case 3

A 39-year-old woman presented with 1-year history of recurrent fever and multiple subcutaneous nodules. A skin biopsy indicated chronic inflammation in the skin and subcutaneous tissue, characterized by the presence of inflammatory cell aggregates. No malignancy was found. The initial diagnosis was subcutaneous panniculitis, for which she received glucocorticoid treatment at a local hospital. However, the subcutaneous nodules continued to recur intermittently. Eventually, she developed liver damage and thrombocytopenia, and was admitted to our hospital. The patient was diagnosed with HLH based on the presence of fever, pancytopenia, decreased fibrinogen levels (0.4g/L), and elevated levels of ferritin(839.10ng/mL), sCD25(60585pg/mL), and triglyceride(10.69mmol/L) (**Table 1**). WES of this patient revealed homozygous missense mutation in the *HAVCR2* gene: c245A > G (p.Y82C). Unfortunately, her condition deteriorated rapidly before receiving treatment, and she passed away due to complications, including skin infection and pneumonia.

### Case 4

**Figure 1:**
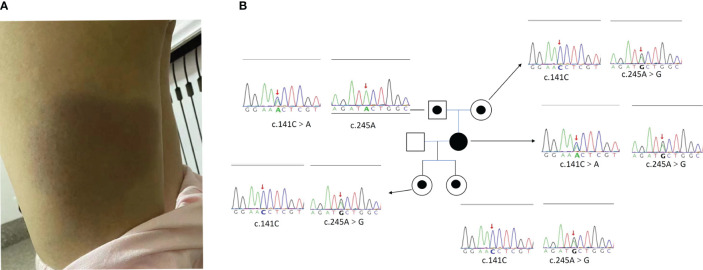
**(A)** The lower limb skin change of Case 4. **(B)** The Pedigree diagram of the proband and corresponding sequencing results. Circles represent female family members, squares represent male family members, and the black small circle represents a single heterozygous mutation in the *HAVCR2* gene. The Sanger sequencing map shows the corresponding mutations in the *HAVCR2* gene for the proband [c245A>G (p.Y82C) and c.141C>A (p.A47L) compound heterozygous mutations] and their family members (with single heterozygous mutation). The red arrow represents the mutation site.

A 39-year-old woman presented with fever and erythema of both lower extremities persisting for 8 months. The affected area exhibited elevated skin temperature. Initially, she was diagnosed with panniculitis by a local hospital and treated with dexamethasone and cephalosporin antibiotics, but her symptoms did not improve. Laboratory tests revealed elevated ferritin levels, ANA titer (1:160), and a positive lupus anticoagulant, leading to a misdiagnosis of undifferentiated connective tissue disease. Subsequently, she received glucocorticoid treatment (specific details unknown), which provided temporary relief. However, after one month, she experienced fever recurrence and worsening of erythema (**Figure 1A**), accompanied by a decrease in blood counts. Further laboratory tests indicated a decrease in PLT to 50*10^9^/L, reduced fibrinogen levels (1.13g/L), and elevated ferritin levels(15084ng/mL) (**Table 1**). PETCT showed that enlargement of lymph nodes in several places throughout the body, splenomegaly, and no tumor was found on pathology of enlarged lymph nodes. She was suspected of HLH and admitted to our hospital. The patient underwent examination by WES, which revealed compound heterozygous *HAVCR2* mutation, c245A>G (p.Y82C) and c.141C>A (p.A47L), inherited from her parents. The family lineage survey map is depicted in **Figure 1B**. The p.A47L TIM-3 is predicted to be benign in silico by Poly Phen-2 (PolyPhen-2 = 0.007) and CADD scores (<10). SIFT considered it as benign (>0.05). However, global minor allele frequency (MAFs) of the p.A47L variant is 0.0024 in the Exome Aggregation Consortium (ExAC) and 0.0001 in the East Asians. Ultimately, the pathogenicity of the p.A47L variant is uncertain based on American College of Medical Genetics and Genomics (ACMG) guidelines (10). However, the pathogenicity of the p.A82C variant has been confirmed by multiple studies (4–6). The prediction scores, MAFs and ACMG ratings for the p.A47L variant and p.Y82C variant are shown in **Supplement Table S1**. Furthermore, the heterozygous p.Y82C mutation has previously been reported in idiopathic HLH (7, 11), and has been shown to increase the risk of HLH (11). Considering the *HAVCR2* mutation and the patient’s clinical manifestations of panniculitis and HLH, diagnosis of *HAVCR2* mutation-associated HLH was made. Upon admission, the patient presented with CNS involvement, characterized by disorientation, cognitive decline, and epilepsy. The patient received treatment with the DEP regimen and achieved complete remission of HLH after four courses of treatment. Subsequently, methylprednisolone maintenance therapy was initiated, and the patient has been followed up for six months with stable condition.

### Case 5

A 33-year-old woman presented with two-year history of fever and subcutaneous abdominal mass. At the onset, there was a decrease in blood cells and an increase in ferritin levels (1682ng/mL). NK cell activity was 2.28%, which was significantly decreased. PETCT scan revealed swelling and increased metabolism in the subcutaneous tissue of the abdomen and buttocks, as well as multiple lymph nodes with increased metabolism, and hepatosplenomegaly. Bone marrow examination showed hemophagocytosis (**Table 1**). The pathological diagnosis of the right buttock and abdomen skin indicated SPTCL. After confirming the diagnosis of SPTCL related HLH and undergoing the HLH94 protocol to control HLH, the patient received four courses of ECHOP chemotherapy (Cyclophosphamide, vinorelbine, doxorubicin liposome, Etoposide, prednisone). During the treatment, there were intermittent subcutaneous nodules and recurrences, leading to the administration of PD-1 monoclonal antibody combined with ICE (Etoposide, Ifosfamide, and carboplatin), which had a poor effect. Additionally, lymphadenopathy was observed once again. Following treatment with PD-1 monoclonal antibody combined with Mitoxantrone and Cedaranilide, the patient’s lymphoma achieved a partial response (PR). Subsequently, the patient presented to our hospital to pursue further treatment through allogeneic hematopoietic stem cell transplantation (alloHSCT). Upon admission, WES examination identified a homozygous mutation of *HAVCR2*, c245A>G (p.Y82C). At present, the patient is waiting for alloHSCT.

## Method

In this study, we reviewed the cytokine test results of five patients. Four patients had cytokine results when HLH was active, and the remaining case had cytokine results with HLH in PR status. Levels of nine cytokines (TNF-α, IL‐6, IL‐10, IFN-γ, IL-RA, IL-17, IL-8, CXCL9, and IL‐18) were measured using the Multifactor Liquid Phase Chip Technology‐Luminex analytical platform system, following the manufacturer’s instructions. Nine healthy controls underwent cytokine testing to help evaluate the degree of cytokine elevation in these patients. Furthermore, we reviewed the cytokine levels of active primary HLH patients in our center over the past three years. A total of nine pHLH patients were included, consisting of three cases of familial hemophagocytic lymphohistiocytosis (FHL)-2 and six cases of familial hemophagocytic lymphohistiocytosis (FHL)-3. Statistical analysis was conducted using GraphPad Prism version 9.0. Non-parametric tests were used to perform pairwise comparisons of cytokine levels among three groups.

## Result

**Figure 2:**
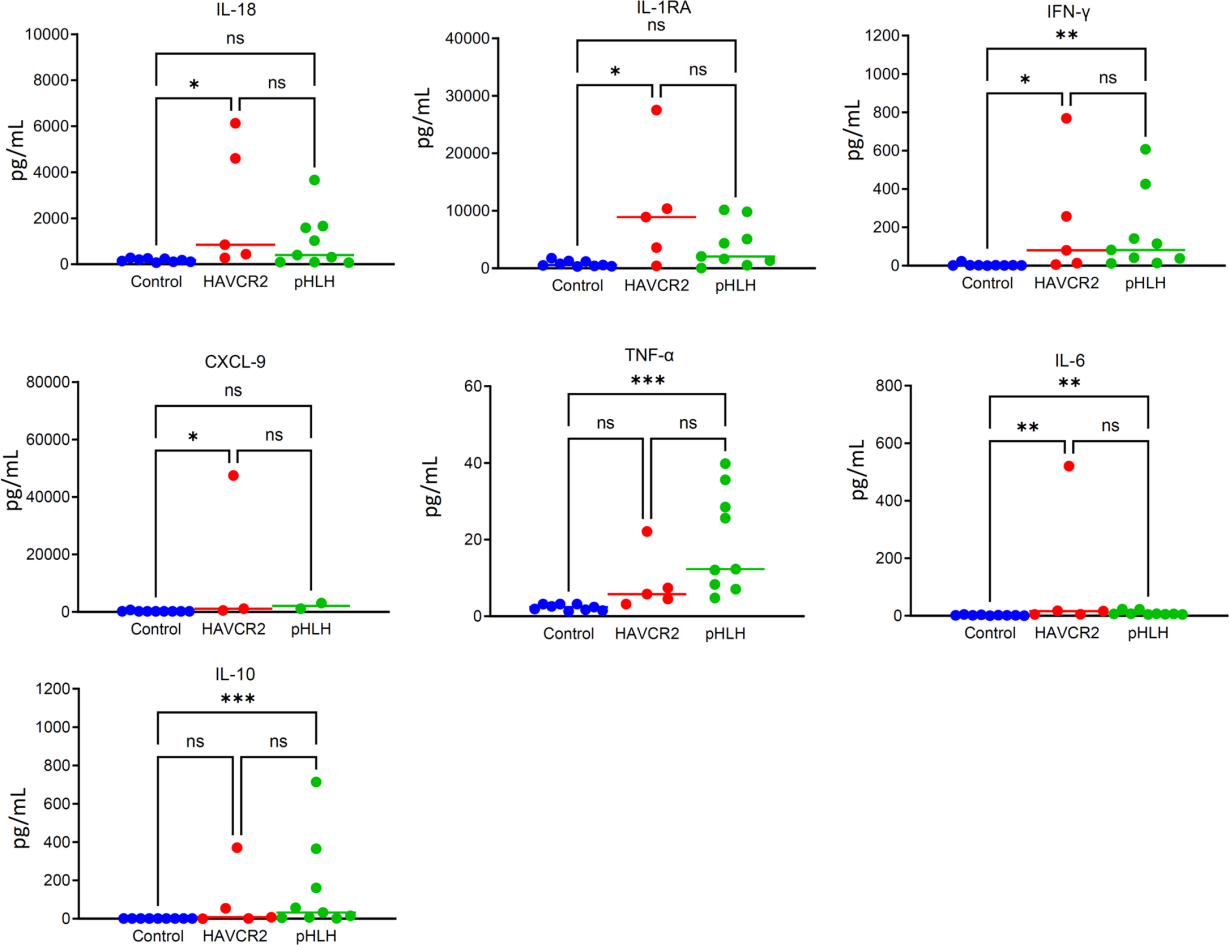
The figure shows the cytokine results of 5 patients with *HAVCR2* mutation-associated HLH, 9 patients with pHLH and 9 healthy controls. The degree of elevation of IL-1RA in the *HAVCR2* mutation-associated HLH group is more significant compared to the control group. IL-1RA levels were higher in the *HAVCR2* mutation-associated HLH group compared to the pHLH group, although there was no statistical difference. The levels of IFN-γ and CXCL9 did not show a significant increase. NS: p> 0.05; *P ≤ 0.05; **P≤ 0.01; ***P≤ 0.001.

The results revealed that, apart from TNF-α, the levels of other cytokines in the *HAVCR2* mutation-associated HLH group were significantly higher compared to the control group, with p-values less than 0.05. There was no significant difference found in the seven cytokines between the *HAVCR2* mutation-associated HLH group and the primary HLH group, which could be attributed to the limited sample size. The distribution of scattered dots revealed that IL-1RA levels were higher in the HAVCR2 mutation-associated HLH group compared to the pHLH group, although there was no statistical difference. Additionally, the levels of IFN-γ and CXCL9 did not show a significant increase. It is important to note that statistical differences may require more patients to confirm. The results are shown in **Figure 2**.

## Discussion

HLH is a serious and fatal disease, presenting mainly as fever, cytopenia, coagulation dysfunction, splenomegaly, and liver dysfunction (1). Previous study showed that approximately 20% of patients with SPTCL developed HLH, which significantly affected their survival (12). The incidence of HLH in patients with SPTCL is significantly higher in those with *HAVCR2* mutation than in those without mutation (4, 5). However, there is also a different view that while the *HAVCR2* mutation frequently occurs in patients with SPTCL, it is not necessarily a significant risk factor for HLH (6). It has been reported that *HAVCR2* mutation can cause HLH even in the absence of lymphoma or panniculitis (11, 13, 14). In this article, three patients also developed HLH without lymphoma, also confirming that *HAVCR2* may be a congenital immune deficiency that triggers HLH. Boonyabaramee et al. have reported that *HAVCR2* mutation-associated HLH is a distinct subgroup of HLH and have better survival (7), which suggested that *HAVCR2* mutation-associated HLH may have a different mechanism than conventional primary HLH. The key mechanism of primary HLH is decreased cytotoxicity, but NK cell degranulation is restored after treatment in patients with *HAVCR2* mutation-associated HLH (13). Further investigation into the mechanisms of *HAVCR2* mutation-associated HLH may contribute to a more comprehensive understanding of HLH mechanisms and potentially identify new therapeutic targets.

**Figure 3:**
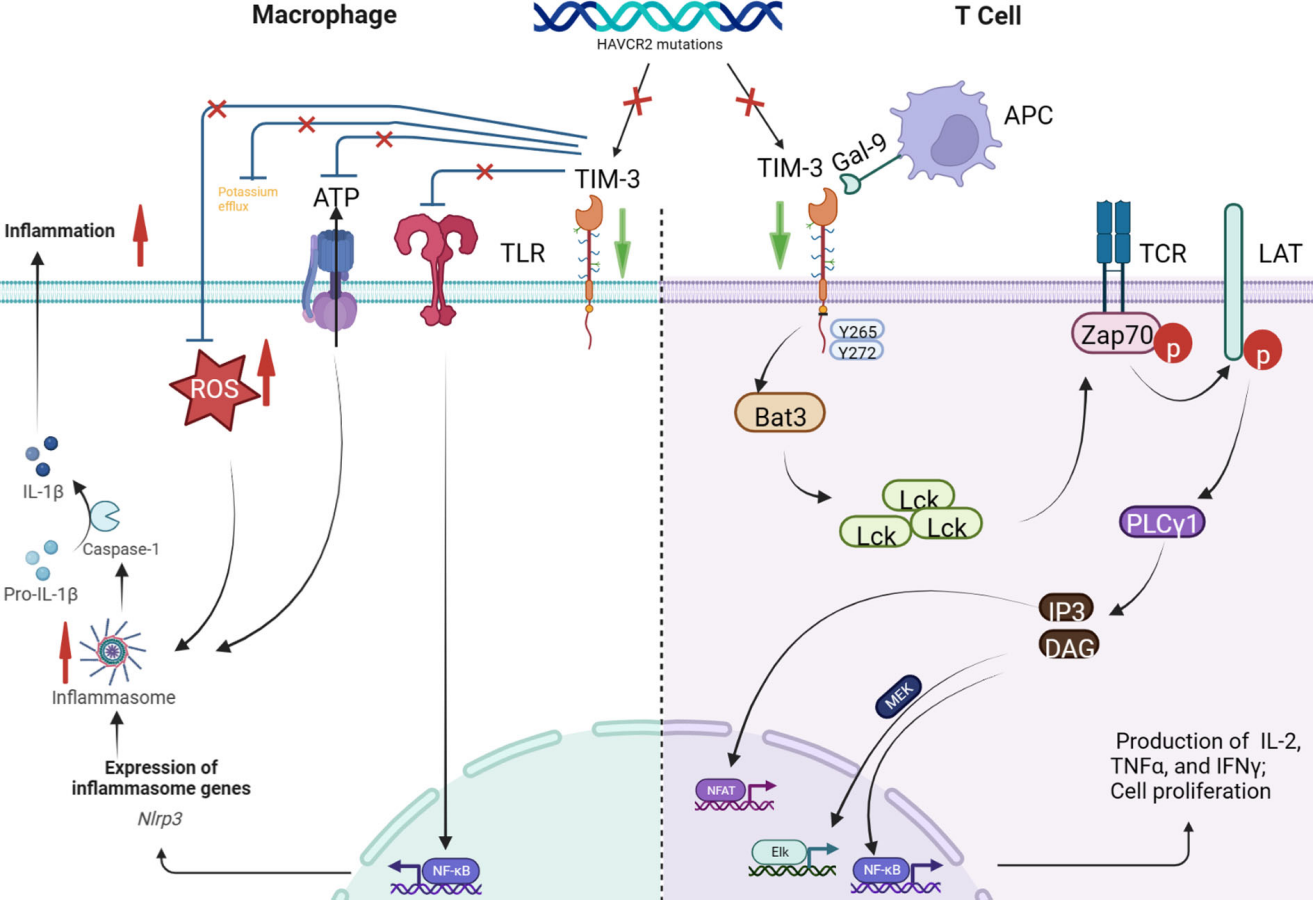
*HAVCR2* encodes TIM-3, a key immune checkpoint inhibitor expressed in T cells, dendritic cells, macrophages, monocytes, natural killer cells, mast cells, and tumor cells (1). A type I membrane protein, TIM-3 inhibits the interferon (IFN)-γ pathway by binding to Galectin-9. *HAVCR2* mutation leads to the loss of Tim-3 expression, blocking the T-cell-mediated inhibition of the IFN-γ pathway, eventually leading to IFN- γ release (2). TIM-3 protein on macrophages inhibits inflammasome activation by inhibiting the TLR-NF-κB pathway, ATP release, K+ efflux, and reactive oxygen species (ROS) production. However, TIM-3 deletion leads to the activation of the TLR-NF-κB pathway and NLRP3 inflammasome in macrophages. The activated inflammasome triggers the autolysis of procaspase-1 to produce activated caspase-1, which cleaves pro-interleukin (IL)-1β and pro-IL-18, producing the corresponding mature cytokine IL-1β and IL-18 to promote the production of IFN-γ and other cytokines. In addition, TIM-3 deletion can also activate the NLRP3 inflammasome by increasing ATP release, K+ efflux, and ROS production.

*HAVCR2* encodes TIM-3, a key immune checkpoint inhibitor expressed in monocytes, lymphocytes, and other immune cells (15). Among the 5 patients we reported, 4 carried germline homozygous mutation of *HAVCR2* mutation (c245A>G; p. Y82C). The homozygous missense variants of *HAVCR2* mutation (c245A > G; p. Y82C) have been reported in East Asian patients with HLH-SPTCL, resulting in misfolding and lowered expression of the TIM-3 protein (4). There are currently reports of germline homozygous *HAVCR2* mutation (c245A>G; p. Y82C) leading to HLH in the absence of lymphoma (11, 13, 14). One possible mechanism of *HAVCR2* mutation-associated HLH involves a decrease in the inhibition of the interferon (IFN)-gamma pathway, resulting in an increase in cytokine release (such as tumor necrosis factor [TNF]-α and IL-1 β) due to decreased TIM-3 expression (15). The serum levels of IFN-γ-induced CXCL10, IL-18, and sCD25 are also significantly higher in HLH-SPTCL patients with *HAVCR2* mutation than in those without mutation. Another potential mechanism involves the activation of the NLRP3 inflammasome and increased release of inflammatory cytokines such as IL-1 β in response to decreased TIM-3 expression. *In vitro* studies indicate that TIM-3 protein loss in the macrophages of patients with HLH-SPTCL and *HAVCR2* mutation lowered the threshold for inflammasome activation and increased the release of inflammatory cytokines (4). One patient achieved HLH remission following treatment with the IL-1 inhibitor anakinra (4). Conditional deletion of TIM-3 in mouse dendritic cells led to an accumulation of reactive oxygen species (ROS) and activation of the NLRP3 inflammasome (16). In a study on panniculitis, TIM-3 protein inhibited the initiation and activation of the NLRP3 inflammasome by inhibiting the TLR-NF-κB pathway, ATP release, K + efflux, and ROS production. Blocking the inhibitory effects of TIM-3 on NLRP 3 inflammasome aggravates peritonitis (17). Therefore, decreased TIM-3 expression in patients with *HAVCR2* mutation leads to panniculitis-like manifestation. **Figure 3** presents the possible mechanisms by which *HAVCR2* mutation may cause HLH.

By comparing cytokines in patients with pHLH and healthy controls, it was observed that patients with *HAVCR2* mutation-associated HLH exhibited an increasing trend in IL-RA levels. This finding suggests that the decrease in TIM-3 expression on macrophages leads to excessive activation of the inflammasome, which could potentially play a significant role in the development of *HAVCR2* mutation-associated HLH. Previous report identified a significant reduction in TIM-3 expression on monocytes in patient with *HAVCR2* mutation-associated HLH (13). Moreover, in SPTCL patients with germline *HAVCR2* mutation, there was a significant decrease in T lymphocytes exerting an inhibitory role (4). Further studies are required to investigate the role of TIM-3 protein in various immune cells in patients with *HAVCR2*-mutation associated HLH.

Gayden et al. proposed that SPTCL-HLH patients with *HAVCR2* mutation benefit from immunosuppressive therapy rather than traditional chemotherapy (4). In this study, two patients improved after treatment with ruxolitinib combined with glucocorticoids or chemotherapy. Zhang et al. also reported favorable efficacy of ruxolitinib in pediatric patients with *HAVCR2* mutation-associated HLH (14). The JAK2 inhibitor, which acts downstream of IFN-γ and other inflammatory cytokine receptors, regulates the JAK1/2 signaling pathway and reduces the expression of various cytokines, thereby controlling HLH (18). Gillard et al. also found that JAK inhibition disrupted T cell-induced macrophage activation and reduced downstream proinflammatory cytokine and chemokine responses (19). The favorable results of ruxolitinib treatment in patients with *HAVCR2* mutation-associated HLH further support the notion that the inflammatory cytokine storm, rather than defective cytotoxicity, plays a significant role in the development of *HAVCR2* mutation-associated HLH.

Notably, although *HAVCR2* mutation-associated HLH can be managed with immunosuppressive therapy, a complete cure can only be achieved through alloHSCT for individuals with germline *HAVCR2* mutation. Hence, it is crucial to maintain regular follow-up to monitor the recurrence of lymphoma and HLH.

## Conclusion

In summary, we reported five cases of HLH associated with *HAVCR2* mutation. Through analysis of the patients’ clinical symptoms and cytokine expression, it was postulated that there may exist a distinct mechanism for *HAVCR2* mutation-associated HLH compared to conventional primary HLH. Further research is necessary to unravel the underlying mechanisms of *HAVCR2* mutation-associated HLH, which may contribute to a more comprehensive understanding of HLH and the identification of potential therapeutic targets.

## Data availability statement

The original contributions presented in the study are included in the article/**Supplementary Material**. Further inquiries can be directed to the corresponding author.

## Ethics statement

The studies involving humans were approved by Ethics Committee of Beijing Friendship Hospital. The studies were conducted in accordance with the local legislation and institutional requirements. The human samples used in this study were acquired from primarily isolated as part of your previous study for which ethical approval was obtained. Written informed consent for participation was not required from the participants or the participants’ legal guardians/next of kin in accordance with the national legislation and institutional requirements. Written informed consent was obtained from the individual(s) for the publication of any potentially identifiable images or data included in this article.

## Author contributions

DS: Data curation, Supervision, Writing – original draft, Writing – review & editing. JW: Supervision, Writing – review and editing. JZ: Writing – review and editing. JH: Writing – review and editing. ZW: Supervision, Writing – original draft, Writing – review and editing. CW: Writing – review & editing.
