# Supplementary material for: Case Report: HAVCR2 mutation-associated Hemophagocytic lymphohistiocytosis
Source: Front Immunol. 2023 Nov 23;14:1271324. doi: 10.3389/fimmu.2023.1271324 (PMC10701531; doi:10.3389/fimmu.2023.1271324)
Supplement: Supplementary file 1 [file Table_1.docx]

**Supplement** **Table S1**. The prediction scores, MAFs and ACMG ratings for the p.A47L variant and p.Y82C variant.

| Variant | MAFs in ExAC | MAFs in East Asians | SIFT prediction | Polyphen-2 prediction | CADD  Score | ACMG rating |
| --- | --- | --- | --- | --- | --- | --- |
| p.A47L variant | 0.0024 | 0.0001 | 0.217(tolerated) | 0.007(benign) | 0.17 | Uncertain |
| p.Y82C variant | 0.0035 | 0.0224 | 0(deleterious) | 0.999(probably damaging) | 23.9 | Pathogenic |
